# Antioxidant Activity and Biocompatibility of Fructo-Polysaccharides Extracted from a Wild Species of Ornithogalum from Lebanon

**DOI:** 10.3390/antiox10010068

**Published:** 2021-01-07

**Authors:** Mohammad Kazem Medlej, Cherri Batoul, Hamza Olleik, Suming Li, Akram Hijazi, Ghassan Nasser, Marc Maresca, Céline Pochat-Bohatier

**Affiliations:** 1 Institut Européen des Membranes, IEM UMR 5635, Université de Montpellier, CNRS, ENSCM, 34095 Montpellier, France; mohammad.medlej1@gmail.com (M.K.M.); batoula.sherry@hotmail.com (C.B.); Suming.Li@umontpellier.fr (S.L.); 2 Platform for Research and Analysis in Environmental Sciences (PRASE), Lebanese University, Beirut, Lebanon; Akram.Hijazi@ul.edu.lb (A.H.); ghassan.nasser@ul.edu.lb (G.N.); 3 Aix Marseille Université, CNRS, Centrale Marseille, iSm2, 13397 Marseille, France; hamza.olleik@live.com (H.O.); m.maresca@univ-amu.fr (M.M.)

**Keywords:** fructo-polysaccharides, ultrasonication, antioxidant activity, biocompatibility

## Abstract

The present study aims to investigate the properties of biopolymers extracted from a Lebanese onion non edible plant. The extraction was performed under mild conditions by varying the percentage of ultra-sound (US) treatment duration to a total extraction time of 30 min (0, 50, 100% US). The extracts were characterized using FTIR, SEC, GC-MS, TGA, and DSC analyses. The composition of the extracts was determined from the total carbohydrate content and protein content measurements. The thermal analyses indicate that all samples have high thermal stability. The antioxidant activities of the extracts were investigated, using β-carotene bleaching, scavenging activity of ABTS, metal chelating ability, and total antioxidant activity tests. The results indicate that the 50% US treatment leads to the best antioxidant activity. Biocompatibility of the extracts was evaluated using hemolysis and cytotoxicity assays. The results showed that 0 and 50% US samples are not toxic to human cells, in contrary to 100% US.

## 1. Introduction

The oxidative process occurs naturally in organisms, leading to the formation of free radicals that can disturb cell wall functions and cause serious DNA damage. Actually, free radicals are associated to many diseases such as arthrosclerosis, cancer, Parkinson’s, Alzheimer’s, etc. [1]. Antioxidants are widely used in pharmaceuticals, food, and the cosmetic industry in order to reduce the harmful effects of free radicals. The antioxidant compounds can be from natural sources or synthetic, however, the latter present many adverse effects such as carcinogenesis and liver injury, especially after long term consumption [2].

Recently, antioxidants from natural sources have attracted more and more attention. In particular, various polysaccharides have been extensively investigated due to their outstanding properties, such as antioxidant [3], anti-tumor, immunological, ant-inflammatory [4], and antidiabetic activities [5].

Different methods are used to extract polysaccharides from plants, including hot water extraction, ultrasound, microwave [6,7], enzymatic hydrolysis, ionic liquids extraction [8], and freeze-thaw extraction [9]. The selected extraction method can affect the antioxidant activity of the products. Chen et al. obtained higher scavenging activity against DPPH for polysaccharides, extracted from *Ornithogalum Caudatum Ait* via ultrasonic, compared to those obtained from heat reflux. In contrast, their scavenging activities against °O_2_^−^ and °OH radicals were lower than those prepared by heat reflux [10]. The extraction conditions also affect the antioxidant activity. Vaquero et al. reported that the ultrasonic amplitude has a negative impact on the DPPH scavenging activity, whereas the extraction time and temperature enhance this activity for polysaccharides extracted from *Laminaria digitate* [11]. Qu et al. reported similar effects of ultrasonic power, extraction time, and temperature on the hydroxyl radical scavenging activity of polysaccharides extracted from *Ziziphus jujuba Mill* [7].

Inulin is a type of fructan, a linear polysaccharide in which the fructose moieties are linked by glycosidic β (2→1) bonds. Shang et al. evaluated the antioxidant activity of inulin and showed that it was weak and significantly lower than those of Vitamin C [12]. In contrast, as a β (2→6) linked fructose polymer, Levan has been used for biomedical applications due to its good antioxidant, anti-inflammatory, and anti-tumor properties [13].

This study reports on the antioxidant properties of a vegetal extract containing mainly polysaccharides and in less extent proteins. The innocuity of the extract was evaluated by conducting hemolysis and cytotoxicity assays on human cells. The extract was obtained from the onion part of *Ornithogalum billardieri*, a wild species harvested in Lebanon, which has never been studied. This study has been conducted to contribute to the development of Lebanon’s natural resources. The polysaccharides were identified in our previous work as fructo-polysaccharides composed of a backbone of (2→6)-linked β-d-fructofuranosyl (Fruf), with (2→1)-linked β-d-Fruf branched chains [14]. Additional GC-MS analyses were subsequently carried out and this paper brings new information about the structure and the composition of the polysaccharides.

The optimum extraction conditions leading to the highest polysaccharides yield and purity were determined using Response Surface Methodology (RSM). The optimal extraction temperature was found to be 44.2 °C. In the present study, new extraction conditions were performed at 25 °C with the aim to reduce energy consumption, but also to evaluate the influence of this parameter on the antioxidant properties of the extract. Three different extraction conditions were applied: maceration (0% US), ultrasonic treatment (100% US), and a combination of both methods (50% US). The antioxidant activities of the extract were investigated from ABTS (2,2-azino-bis (3-ethylbenzothiazoline-6-sulfonic acid)) radical scavenging activity, total antioxidant capacity, metal-chelating power, and *β*-carotene bleaching tests. The antioxidant activity of the different extracts (0, 50, and 100% US) obtained at 25 °C was evaluated in comparison with samples extracted under the optimal conditions at 44.2 °C, denoted OP%US, extracted at 44.2 °C.

The biocompatibility of the extract was examined by hemolysis test and cell viability assay using human normal cells (i.e., HaCaT, HUVEC, and IMR90 cells). The results are reported herein in comparison with those of the extract denoted OP%US obtained under the optimum extraction conditions determined by RSM.

## 2. Experimental Part

### 2.1. Materials and Reagents

Sulfuric acid, phenol, 2,2-azino-bis (3-ethylbenzothiazoline-6-sulfonic acid) (ABTS), ascorbic acid, potassium peroxydisulfate, sodium phosphate, ammonium molybdate, α-tocopherol, iron dichloride, ferrozine, diethylenediamine (EDTA), phenol, pullulan, anhydrous ethanol, butanol, and chloroform of analytical grade were purchased from Sigma Aldrich (Lyon, France), and used as received.

### 2.2. Extraction

The collected plant was first purified using Soxhlet, as previously reported [14]. 1 g of the resulting powder was mixed to 10 mL of ultrapure water. The mixture was homogenized for 30 s at 3500 rpm using a speed mixer (Hauschild DAC 150.1 FVZ-K). The extraction was carried out at 25 °C during 30 min. The extraction temperature was controlled (±0.2 °C) using a thermo-cryostat (Vacuo-Temp P, Selecta, Barcelona, Spain) and a thermostat cell. Ultrasound (Badelin SonoRex, Berlin, Germany) treatment was performed at fixed frequency (35 KHz) and power (120 W). The time ratio of ultrasound treatment to the total duration of the extraction process (%US) was 0, 50, and 100%. After extraction, the aqueous solution was centrifuged at 6000 rpm for 30 min. The supernatant was precipitated in 90% ethanol, and the extract was collected after centrifugation at 6000 rpm. Finally, the obtained product was vacuum dried up to a constant weight. The extraction yield (%) was calculated using Equation (1).

Extraction yield (%) = (Weight of dried product/Weight of crude powder) × 100(1)

### 2.3. Characterization of the Extracts

#### 2.3.1. Total Carbohydrates Content (TCC)

The TCC was determined according to sulfuric acid-phenol method also called Dubois method [15]. A stock solution of extract at 2.5 mg/mL was prepared, and diluted 200 times. 500 µL of the diluted solution was deposited in a tube with 2.5 mL of concentrated H_2_SO_4_ (95–99%) and 500 µL of phenol (5% *w*/*w*). The tube was incubated for 10 min at 100 °C. After 20 min in the dark at room temperature, the absorbance was measured at 490 nm. A standard curve of glucose at concentrations from 0 to 100 μg/mL was previously established in the same conditions [15]. The TCC % was calculated using the following equation.

TCC (%) = [(Weight of polysaccharides/Weight of sample)] × 100(2)

#### 2.3.2. Protein Content

The protein content was estimated according to the Lowry method, using BSA (bovine serum albumin) as standard [16]. A stock reagent solution was first prepared by mixing 2% (*w*/*v*) Na_2_CO_3_, 1% (*w*/*v*) CuSO_4_, 5H_2_O, and 2% (*w*/*v*) sodium potassium tartrate in volume ratio of 100:1:1. Then, 0.1 mL of 2N NaOH was added to 0.1 mL of sample solution, and the mixture was heated at 100 °C for 10 min in boiling water bath. The mixture was cooled down to room temperature and 1 mL of freshly prepared reagent was added, followed by addition of 0.1 mL of Folin reagent (1N) 10 min later. After homogenization, the solution was allowed to stand at room temperature for 30 min, and the absorbance was measured at 550 nm. A standard curve was previously established by using bovine serum albumin (BSA) at different concentrations ranging from 0.1 to 1 mg/mL [16].

#### 2.3.3. Fourier Transform Infrared (FTIR) Spectroscopy

ATR–FTIR spectra were obtained on Nicolet-5700 FTIR spectrometer with attenuated total reflection (ATR) using ZnSe crystal diamond. 32 scans were recorded at 4 cm^−1^ resolution over a range of 650–4000 cm^−1^.

#### 2.3.4. Size-Exclusion Chromatography (SEC)

Size-exclusion chromatography (SEC) was carried out using HPLC (DW-LC1620A) equipped with TSK gel PW5000 + PW3000 columns and refraction index and ultraviolet detectors. The temperature of the columns and detectors was set at 20 and 35 °C, respectively. A pH 6 phosphate buffer at 10 mg/mL was used as eluent at a flow rate of 1 mL/min. Calibration was realized using pullulan standards with molar masses from 500 to 25,000 Da. The results were processed using OmniSEC software.

#### 2.3.5. Monosaccharide Composition and Linkage Type Analysis

The monosaccharide composition of the extract was determined according to the method of Montreuil et al. (1986). The neutral sugars composition was identified as trimethylsilyl derivatives after acidic methanolysis and subsequent GC–FID analysis [17]. 50 μg of sample were added to 500 μL of an ahydrous solution of methanol/3N HCl, together with 50 μg of pentaerythritol and 50 μg of myo-inositol as internal reference. After 18 h at 80 °C, the solution was cooled down to room temperature, and the methanolysate was neutralized using silver carbonate AgCO_3_. Then 20 μL of acetic anhydride were added in order to reacetylate the osamines. After overnight, in the dark at room temperature, the solution was centrifuged for 15 min at 4000 rpm, and the supernatant was evaporated under nitrogen atmosphere.

The obtained trimethylsilylated methylglycosides were added to a mixture of 1 mL of MeOH and 1 mL of heptane, and homogenized. The resulting suspension was then centrifuged 3 times. After drying, the precipitate was added to a mixture of 10 µL of pyridine and 10 µL of N, O Bis(trimethylsilyl)trifluoroacetamide (BSTFA). After vigorous stirring for 2 h, the solution was analyzed by Gas Chromatography—Mass Spectrometry (GC–MS). GC–MS analysis was performed on an AGILENT GC-7820A system (in-column injection, FID detector: flame ionization) using hydrogen as carrier gas and 1D-SolGel-1MS 0.25 µm type column.

According to the method of Hakomori, the analysis of the glycosidic bonds was carried out by partially methylated and acetylated derivatization (PMAA method—partially methylated and acetylated alditols method) [18]. The PMAA protocol uses DMSO and dimsyl sodium as base and methyl iodide as a methylating agent. The dimsyl ion is generated by mixing 20 mg of NaH with 200 µL DMSO. Next, 100 µL of solution was added to 200 µg of sample with 100 µL of ICH_3_. After 15 min of stirring, 2 mL of CHCl_3_ were added to the mixture. The CHCl_3_ layer was washed 4 times with 2 mL of H_2_O. The aqueous phase was then removed, and the CHCl_3_ phase was evaporated under nitrogen. The hydrolysis of the product was carried out by adding 500 µL of 4 M trifluoroacetic acid at 100 °C for 4 h. After drying, 500 µL of sodium borodeuteride were added, and stirred vigorously for several minutes. Then, the solution was dried under nitrogen flow and put under a desiccator overnight.

The pellet was then washed with anhydrous MeOH 4 times, followed by solvent evaporation and vacuum drying. The peracetylation reaction was then carried out at 100 °C for 4 h after addition of 10 µL of pyridine and 500 µL of acetic anhydride. The solution was then dried. 500 µL of water were added to the pellet, and washed with CHCl_3_ 4 times. After removal of the aqueous phase, the CHCl_3_ phase was dried. Finally, 500 µL of CHCl_3_ were added to the product. Then, 1 µL was injected into the GC–FID and diluted a tenth before injection in GC–MS. Saccharose was used as a standard for both analyses, and myo-inositol was used as internal reference.

#### 2.3.6. Thermogravimetric Analysis (TGA)

Thermogravimetric analysis was carried out using Q-500 apparatus (TA Instruments). The sample was heated over the temperature range of 25 to 800 °C at a rate 20 °C/min under nitrogen purge.

#### 2.3.7. Differential Scanning Calorimetry (DSC)

Differential scanning calorimetry was performed using Q-20 apparatus (TA Instruments). The sample was heated in a temperature range between −80 °C and 200 °C with a ramp of 10 °C/min. A nitrogen flow of 50 mL/min was applied during analyses. Two heating cycles were performed for each sample.

### 2.4. Evaluation of Antioxidant Activities

#### 2.4.1. ABTS Radical Cation Decolorization Assay

ABTS (2,2′-azino-bis (3-ethylbenzo-thiazoline-6-sulfonic acid) forms a relatively stable free radical, which decolorizes in its non-radical form. First, 7 mM of ABTS were dissolved in water with 2.45 mM K_2_S_2_O_8_. The mixture was stirred in the dark at room temperature for 12 h in order to generate a cationic radical ABTS^+^. The solution was diluted in ethanol. An aliquot of 0.1 mL of samples at concentrations ranging from 1 to 10 mg/mL is mixed with 3.9 mL of the as-prepared solution of ABTS^+^. After incubation at 30 °C for 20 min, the absorbance is measured at 734 nm. Control is prepared by replacing the sample with ultrapure water [19]. The scavenging rate is calculated according to Equation (3).

Scavenging rate (%) = [A_0_ − (A_e_ − A_ie_)]/A_0_ × 100 (3)

where A_0_ is the absorbance of blank sample, A_e_ is the absorbance of samples, and A_ie_ is the absorbance of positive control.

#### 2.4.2. β-Carotene-Linoleic Acid Assay

The antioxidant capacity of samples was determined according to the method of β-carotene-linoleic acid with slight modification. This test allows determining the capacity of the extracts to neutralize free lipophilic radicals by inhibition of oxidative degradation of β-carotene.

An emulsion of β-carotene and linoleic acid was first prepared by dissolving 0.5 mg of β-carotene, 25 μL of linoleic acid, and 200 µL of Tween 80 in 1 mL of chloroform. The solvent was then completely evaporated using a rotary evaporator at 45 °C. Then, 100 mL of ultrapure water was added under stirring to yield a suspension, and 2.5 mL of freshly prepared suspension were transferred to tubes containing 0.5 mL of test samples at concentrations ranging from 0.05 to 5 mg/mL. The mixture was incubated for 2 h at 50 °C after homogenization. Finally, the tubes were placed in a water bath at room temperature. The absorbance was measured at 470 nm [20]. Butylated hydroxyanisole (BHA) was used as positive control. The antioxidant activity was evaluated using the following equation.

Inhibition (%) = [1 − (A_t0_ − A_t120_) _sample_/(A_t0_ − A_t120_) _control_] × 100 (4)

where A_t0_ and A_t120_ are the absorbance of the sample or the control before and after 120 min incubation.

#### 2.4.3. Ferrous Ion-Chelating Activity

The chelating ability was evaluated by measuring the decolorization of complex (Fe^2+^ ferrozine) at 562 nm. 100 μL of samples at concentrations ranging from 0.2 to 10 mg/mL were mixed with 50 µL of FeCl_2_ (2 mM). After vigorous shaking, the solutions were allowed to stand for 5 min. Then 100 µL of 5 mM Ferrozine and 2.75 mL of ultrapure water were added under stirring. After 10 min of incubation, the absorbance was measured at 562 nm [21]. The negative control was prepared without the addition of the test sample, and EDTA was used as the positive control.

The antioxidant activity was calculated from the following equation:

Antioxidant activity % = [(A_control_ − A_sample_)/A_control_] × 100 (5)

where A_control_ and A_sample_ are the absorbance of the negative control and the sample, respectively.

#### 2.4.4. Total Antioxidant Activity Assay

The total antioxidant activity of the extracts was evaluated according to the method described by Prieto et al. [22]. First, 100 µL of samples at concentrations ranging from 0.025 to 10 mg/mL were added to 1 mL of reagent solution (0.6 M sulfuric acid, 28 mM sodium phosphate and 4 mM ammonium molybdate). After incubation at 95 °C for 90 min, the mixture was cooled down to room temperature, and the absorbance was measured at 820 nm. Ultrapure water was used as control. The antioxidant activity is presented in terms of absorption.

### 2.5. Cytotoxic Assay on Human Cells

The toxicity of the extracts on human cells was evaluated using resazurin assay, as previously described [23,24]. Human cells used were HaCaT (Creative Bioarray, Shirley, NY 11967, USA), HUVEC (ECACC, Sigma–Aldrich, Lyon, France), and IMR90 cells (ATCC^®^ CCL186) (ATCC, Molsheim CEDEX, France) corresponding to normal human epidermal keratinocytes, primary human umbilical vein endothelial cells, and normal human lung fibroblast, respectively. HaCaT and IMR90 cells were cultured in Dulbecco’s modified essential medium (DMEM) supplemented with 10% fetal bovine serum (FBS), 1% L-glutamine, and 1% antibiotics (all from Thermo Fisher Scientific, Illkirch–Graffenstaden, France), whereas HUVEC cells were cultured in endothelial specific medium (Sigma–Aldrich, Lyon, France). Cells were routinely grown on 25 cm^2^ flasks and maintained in a 5% CO_2_ incubator at 37 °C. For toxicity assay, cells were detached using a trypsin–EDTA solution (Thermo Fisher Scientific, Illkirch–Graffenstaden, France), counted using Mallasez counting chamber and seeded into 96-well cell culture plates (Greiner bio-one from Dominique Dutscher, Brumath, France) at approximately 10,000 cells per well. The cells were allowed to grow for 48–72 h at 37 °C in a 5% CO_2_ incubator up to confluence. The medium was then aspirated and the cells were treated with 100 µL of culture media containing samples with increasing concentrations obtained by serial dilution (from 0 to 20 mg/mL, 1:2 dilution). After 48 h incubation at 37 °C in a 5% CO_2_ incubator, the wells were emptied, and the cells were treated with 100 µL of resazurin diluted by 1:10 in sterile phosphate-buffered saline (PBS) containing calcium and magnesium (PBS^++^, pH 7.4). After 4 h incubation at 37 °C, the fluorescence intensity was measured at excitation wavelength of 530 nm and emission wavelength of 590 nm, using a microplate reader (Biotek, Synergy Mx, Colmar, France). The fluorescence values were normalized by the negative control corresponding to cells receiving only vehicle (DMSO treated cells using maximal final concentration 1% *(v/v)*), and were expressed as percent viability. The IC_50_ values of the samples on cell viability (i.e., the concentration of test samples causing 50% apoptosis) were calculated using Grap

hPad^®^ Prism 7 software (San Diego, CA, USA). Experiments were conducted in triplicate (*n* = 3).

### 2.6. Hemolytic Activity Assay

The hemolytic activity of the extracts was determined from the leakage of hemoglobin from human erythrocytes, as previously described [25,26]. Briefly, human erythrocytes (red blood cells, RBC) (Divbioscience, NL) were washed twice with sterile PBS, pH 7.4 (Thermo Fisher Scientific, Illkirch–Graffenstaden, France), and pelleted using centrifugation at 800× *g* for 5 min. Human erythrocytes were then suspended in PBS at a final concentration of 8%. 150 μL of the suspension were then added in each well of sterile 96 well microplates (Greiner bio-one from Dominique Dutscher, Brumath, France), and exposed to extract solutions at increasing concentrations (from 0 to 20 mg/mL, 1:2 dilution). After 1 h at 37 °C, the microplates were centrifuged at 800× *g* for 5 min. Then, 100 μL of supernatant were carefully collected and transferred to a new 96 well microplate. The optical density (OD) was measured at 405 nm using microplate reader (Biotek, Synergy Mx, Colmar, France). The hemolytic activity was expressed as percentage of hemolysis, using Triton-X100 at 0.1% (*v*:*v*) as positive control which causes 100% hemolysis. The HC_50_ values of the tested samples (i.e., the concentrations causing 50% of hemolysis) were calculated using GraphPad^®^ Prism 7 software (San Diego, CA, USA). Experiments were conducted in triplicate (*n* = 3).

### 2.7. Statistical Analysis

All experiments were run at least in triplicate. Data are expressed as mean ± SD (standard deviation). A one-way analysis of variance (ANOVA) was then performed and allows estimating the significance. A value of *p* < 0.05 is considered statistically significant.

## 3. Results and Discussion

The samples obtained by 0, 15, and 30 min sonication, for a total extraction time of 30 min, are denoted 0, 50, and 100% US.

### 3.1. Extraction Yield of Polysaccharides and Composition of the Extract

Table 1 presents the extraction yield, TCC, and proteins content data of the extracts obtained under different ultrasound treatments (0, 50, and 100%). Data are compared to those of the extract obtained under the optimal extraction conditions (OP%US) determined in our previous work using Surface Response Methodology (extraction time: 37.1 min, temperature: 44.2 °C, water volume to mass ratio: 33.8 mL/g, and US%:51.7%) [14]. The results indicate that the samples obtained with 50% US exhibit higher extraction yield (45.5%) than the samples with 0% US (25.5%) and 100% US (37.9%). The same trend is observed for TCC (%) which increases from 58.2% for 0% US, to 80.2% for 50% US, and decreases to 66.1% for 100% US. The OP%US actually presents the best extraction yield (85.7%) and TCC (83%) compared to 0, 50, and 100% US extracts. This finding can be attributed to the milder extraction conditions used in this work (25 °C, 30 min, and 10 mL/g) compared to those of OP%US. Anyway, both extracts (OP%US and 50% US) give evidence that the combined treatment improves the polysaccharide extraction by leveraging ultra-sonic treatment. Indeed, sonication destroys the plant cell walls and reduces the particle size which enhances the polysaccharides extraction [27]. Nevertheless, excessive US treatment induces chain cleavage and destruction of polymer chains due to the cavitation effects [28].

Besides, the results show that, under mild temperature (25 °C), increasing US treatment from 0 to 100% leads to protein content increase from 2.4 to 7.7%.

Hou et al. reported also that the ultrasound enhances proteins extraction from Chestnut. Extraction was made at 80 °C for 30 min with a ratio volume to mass ratio of 20 mL/g. The content of proteins in the crude extracts is 1.9 and 3.7%, without or with ultrasound treatment [29].

Therefore, the effect of ultrasound on the breaking of the plant cell walls also favors proteins extraction.

It is noteworthy that the sum of TCC and proteins content is largely below 100, even taking into consideration the presence of residual water as determined by thermogravimetric analysis (TGA) (Table 1). This finding suggests that there are other components in the extracts. However, the 50% US extract is clearly purer than the 0and 100% US extracts as it has much higher TCC of 80.2%.

### 3.2. FTIR

**Figure 1:**
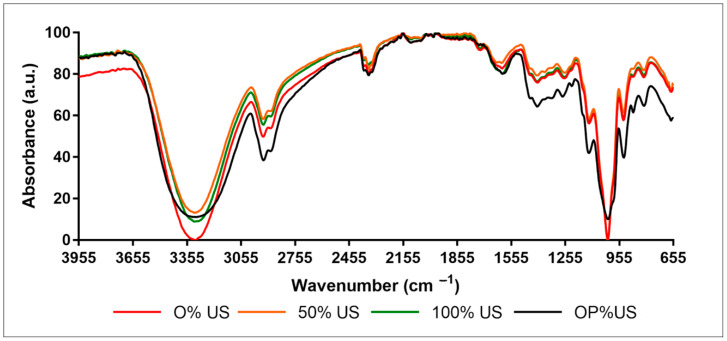
FTIR spectra of 0, 50, 100, and OP%US extracts.

Figure 1 presents the FTIR spectra of the three extracts, 0, 50, 100, and OP%US.

The IR spectra show a peak of stretching vibration around 3317 cm^−1^, indicating the presence of O-H into the extracts. The two peaks detected at 2937 cm^−1^ and 2889 cm^−1^ are associated to −CH_3_ and −CH_2_ groups, respectively. The large band detected in the region of 1580–1720 cm^−1^ can be related to the primary and secondary amine and amide [30]. The peak at 1650 cm^−1^ is attributed to the carboxyl group and N–H asymmetric vibration [31]. The absorption peaks at 1650 cm^−1^ and 1417 cm^−1^ are characteristic of protein absorption [32]. The fingerprint of polysaccharides is monitored by the presence of two strong bands at 1130 cm^−1^ and 1027 cm^−1^ assigned to C–O–C stretching of glycosidic bond [33]. The band detected at 931 cm^−1^ is attributed to the symmetric stretching of furanose rings. The characteristic absorption at 813 cm^−1^ indicates the presence of alfa configuration in the polysaccharides.

The three samples present the same characteristic bands. Nevertheless, some intensity differences are detected. After normalization and base line correction, the surface areas between 1500 and 1712 cm^−1^ of each spectrum were calculated using LabSpec V.5.45.09 software. The surface areas values are 1.19, 1.30, 1.67, and 0.9 a.u. for 0, 50, 100% US, and OP%US, respectively. The surface areas between 1500 and 1712 cm^−1^ change well corroborates with the rising of protein content with the % US (Table 1).

### 3.3. Size-Exclusion Chromatography

The weight average molar mass (Mw) and the dispersity (Ð) of the extracts were determined by SEC in an aqueous medium (Table 1). Little difference is detected between the Mw and Ð values of the three samples. The results indicate that an increase in ultrasound percentage has only little effect on the polysaccharides molar mass, probably due to the mild extraction conditions (25 °C, 30 min, 10 mL/g).

### 3.4. GC–FID of TMS Derivatives Analysis

As shown in the chromatogram (Figure S1), the methanolysis of sucrose (α-Glc- (1-2) -β-Fru) yields glucose in the form of alpha and beta methylglucopyranoside derivatives at retention times of 39.0 and 37.8 min, respectively. Isomerization of fructose leads to alpha and beta methylgluco-pyranosides overlapped with methyl-glucopyranosides derived from glucose, and little amount of methyl-fructofuranosides (retention time 42.8 min). This observation prohibits the quantification of residues.

As shown in Figure S2, the methanolysis of OP%US gives two expected monosaccharide derivatives in proportions that do not represent the stoichiometry of the original molecule (isomerization of fructose residues during methanolysis). Thus, the beta-methyl-per-silyl-glucopyranoside derivatives are detected at 37.73 min and the beta-methyl-per-silyl-fructopyrannosides are detected at 42.82 min. Taking into consideration the poor response of fructose in its native form, the peak at 42.82 min indicates a high proportion of fructose residues.

### 3.5. GC–MS of PMAA Derivatives Analysis

**Figure 2:**
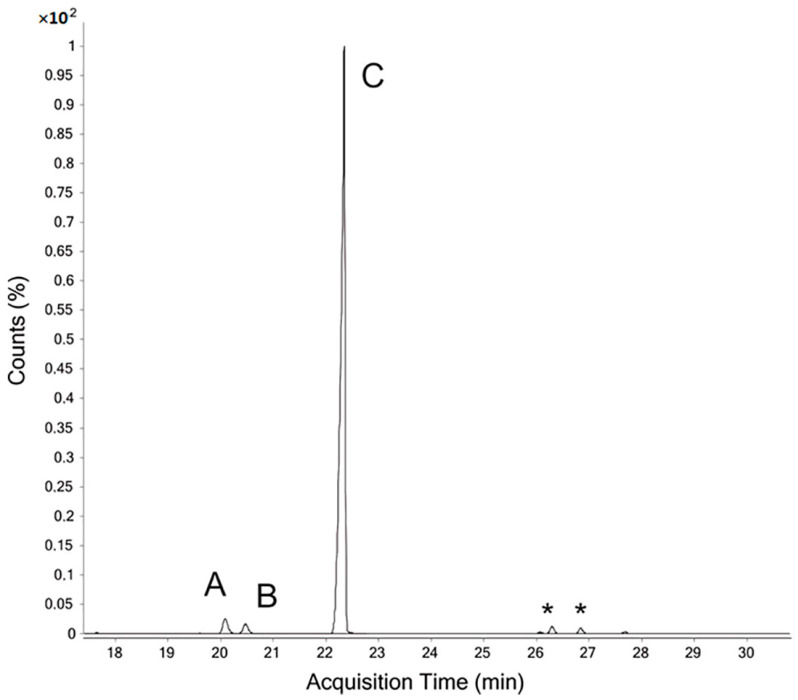
TIC chromatogram of PMAA derivatives obtained from sucrose (* disubstituted derivatives of glucose derived from starch, intrinsic contamination).

The chromatogram of the sucrose analysis shows three main peaks A, B, and C at 20, 20.5, and 22.3 min, respectively (Figure 2). The attribution of those peaks was carried out after analyzing the mass spectra obtained by electronic impact. The peaks A and B present exactly the same fragmentation spectra (Figures S3 and S4). The fragmentation rules show the ketotic origin of this compound. Actually, methylation on carbon 1 and deuteration as well as acetylation on carbon 2 indicate that this is a derivative of fructose. The absence of the *m/z* 45, 118, and 205 fragments, and the co-presence of the *m*/*z* 161 and 162 ions, allow to confirm that the peak A is a residue of 2,5-di-O-acetyl-1,3,4,6 tetra-O-methyl-hexitols. Similarly, the peak B shows the presence of derivative of the ketosis residue attributed to fructose, as evidenced by the fragmentation spectrum (Figure S4). The two retention times therefore suggest the presence of two isomers resulted from the isomerization of the fructose residue (i.e., 2-ketose) during hydrolysis and reduction. Carbon 2 has been reduced either with an R or S configuration. This residue therefore gives, after reduction, 1,3,4,6 tetra-O-methyl-glucitol and 1,3,4,6 tetra O-methyl-mannitol (C-2 epimer). Meanwhile, the major ions *m/z* of peak C determined from the chromatogram fragmentation are 118 and 205 (Figure S5), indicating clearly that they are derived from an aldose residue, and can be attributed to sucrose glucose. This fragmentation spectrum corresponds to 2,3,4,6tetra-O-methyl-1,5-di-O-Acetyl-Glucitol.

**Figure 3:**
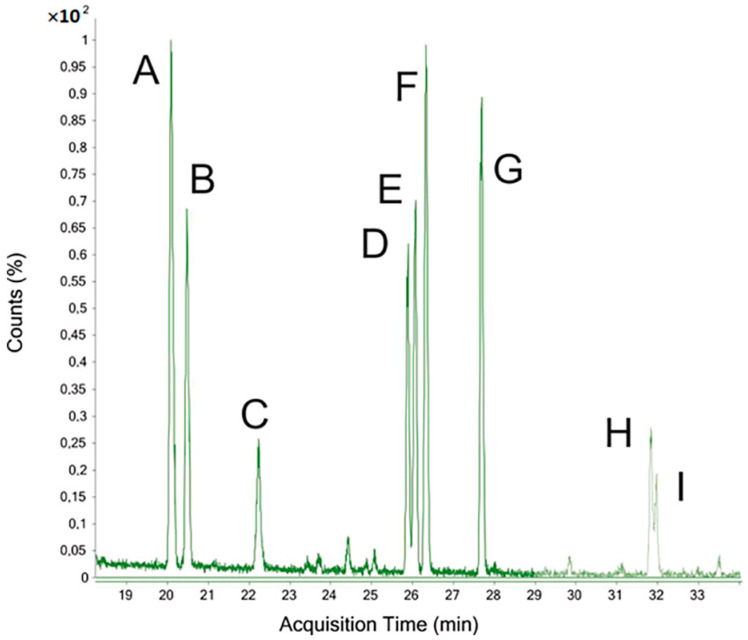
TIC chromatogram of PMAA derivatives obtained for the sample OP%US.

The total ion chromatogram (TIC) obtained from GC–MS of the OP%US (Figure 3) indicates the presence of 9 peaks, i.e., A-I.

The peaks A and B are assigned to 2,5-di-O-acetyl-1,3,4,6 tetra-O-methyl-glucitol, and 2,5-di-O-acetyl-1,3,4,6 tetra-O-methyl-mannitol, respectively. Therefore, they are derived from unsubstituted terminal fructose residues. The peak C is attributed to 2,3,4,6 tetra-O-methyl-1,5-di-O-Acetyl-Glucitol obtained from unsubstituted terminal glucose residues (primer of inulin). These three peaks correspond to the di-acetyl residues in the non-reducing terminal position.

The peaks D (Figure S6) and E (Figure S7) are identified as residues of 2,5,6-tri-O-Acetyl-1,3,4-tri-O-methyl-Hexitol and 1,2,5, tri-O-Acetyl-3,4,6-tri-O-methyl-Hexitol, respectively. The two components are derived from fructose substituted in 6 and 1 and therefore from glucitol.

Peak F (Figure S8) is assigned to a mixture of the two residues D and E which have been subjected to steroisomerization during the reduction and correspond to PMAA residues of mannitol. The two PMAA molecules exhibit identical stereochemistry (axis of symmetry of the chiral carbons). Their retention time is the same. These three peaks therefore represent the tri-O-acetylated residues of fructose monosubstituted in 1 or 6.

The fragmentation study of the peak G (Figure S9) in electronic impact shows that this peak is a derivative of 1,5,6-tri-O-acetyl-2,3,4-tri-O-methyl-hexitol, demonstrating the presence of a substituted aldopyranose in position 6. This could characterize a 6-substituted glucose residue originating from neo-inulin or neo-levan (e.g., neokestose) [34].

The mass spectrometry fragmentation of two peaks H and I present exactly the same spectra of electronic impacts (Figure S10). The *m*/*z* ions are detected at 189 and 190, together with their daughter ions detected at 129 and 130 that indicate a loss of acetic acid. The absence of *m/z* ions of 45 is also noted. The spectrum clearly indicates that the peaks H and I are 3,4-di-O-Methyl-1,2,5,6-tetra-O-acetyl-hexitols residues. These residues prove the existence of hexoses di-substituted in 1 and 6. The different retention times of these same molecules are assigned to the isomerization during hydrolysis and reduction in ketose (fructose), thus yielding di-Me-tetra-O-Ac-mannitol and di-Me-tetra-O-Ac-glucitol.

The compositional analysis clearly indicates that OP%US is exclusively composed of fructose in large quantities and of glucose. The analysis of the bonds between the various monosaccharides shows that there are numerous residues of fructose monosubstituted in 1 or 6 and of glucose in position 6. The compounds di-substituted in 1 and 6 have also been identified, indicating the presence of branches on fructose. These compounds belong to the family of inulins, neo-inulins, levans, neo-levans, and graminane.

### 3.6. Thermal Analysis

**Figure 4:**
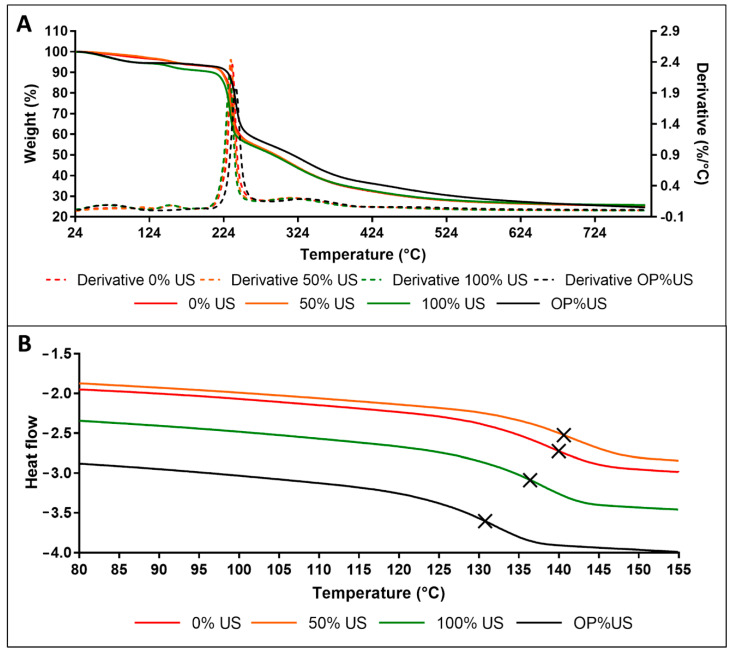
TGA curves (**A**), second heating scan thermograms of DSC (**B**) of 0, 50, 100% US and OP%US.

The TGA thermograms of the three extracts are presented in Figure 4A. A slight weight loss of 3.4, 3.0, and 5.6% is detected from 25 to 125 °C for 0, 50, and 100% US, respectively. While a loss of 5% is detected from 25 to 100°C for OP%US. Then, another small weight loss of 3.1, 2.9, and 2.7% is detected between 125 and 180 °C for 0, 50, and 100% US samples, respectively. The first decrease is attributed to free water evaporation, and the second one to the evaporation of bound water [35]. Beyond, the three curves almost overlap. A weight loss of 41.8% is detected from 200 to 287 °C, followed by a loss of 17.6% from 287 to 404 °C. While for OP%US a weight loss of 35.2% is detected from 224 to 262 °C, followed by a loss of 19.9% from 262 to 404 °C.

The derivative curves show two maxima of degradation rate at 235.6 and 330.8 °C. These two phases correspond to the thermal degradation of two types of ramification. In fact, β (2→1) branch linkages are first broken, followed by main chain β (2→6) linkages of pyranose rings [36,37]. Finally, a weight loss of 8.4% is detected between 404 and 800 °C due to the degradation and pyrolysis of the backbone. These findings evidence the high thermal stability of the extracts below 200 °C.

Figure 4B presents the DSC thermogram of 0, 50, and 100% US. The glass transition temperature (Tg) of the extracts was determined from the second heating scan as the first scan allowed to erase the thermal history. Tg is very close for 0 and 50% US (140.6 and 139.9 °C, respectively). A slightly lower Tg of 136.5 °C is obtained for 100% US. Tg is 130.7 °C for OP%US, showing a decrease of about 9 °C compared to 50% US, and meaning that the effect of temperature is more significant in these conditions than the percentage of ultrasonic treatment.

Anyway, globally, both thermal analyses displays similar results for the three extracts obtained at 25 °C, and support the first conclusions based on FTIR and SEC investigations. The impact of the % of ultra-sound treatment on the chemical composition of the three extracts is quite low.

### 3.7. Antioxidant Activities

The antioxidant activity of the different extracts (0, 50, and 100% US) obtained at 25 °C was evaluated in comparison with OP%US, extracted at 44.2 °C. Four tests were selected according to their mechanisms of action. Firstly, the reduction in molybdenum Mo (VI) was used to evaluate the total antioxidant capacity of the extracts [22]. β-carotene bleaching inhibition was used to evaluate lipophilic radical inhibition of the extract, ABTS assay to evaluate the inhibition capacity of hydrophilic radicals, and ferrous chelating to determine the ability of extracts to reduce the generation reaction of radicals.

#### 3.7.1. Evaluation of Total Antioxidant Capacity

**Figure 5:**
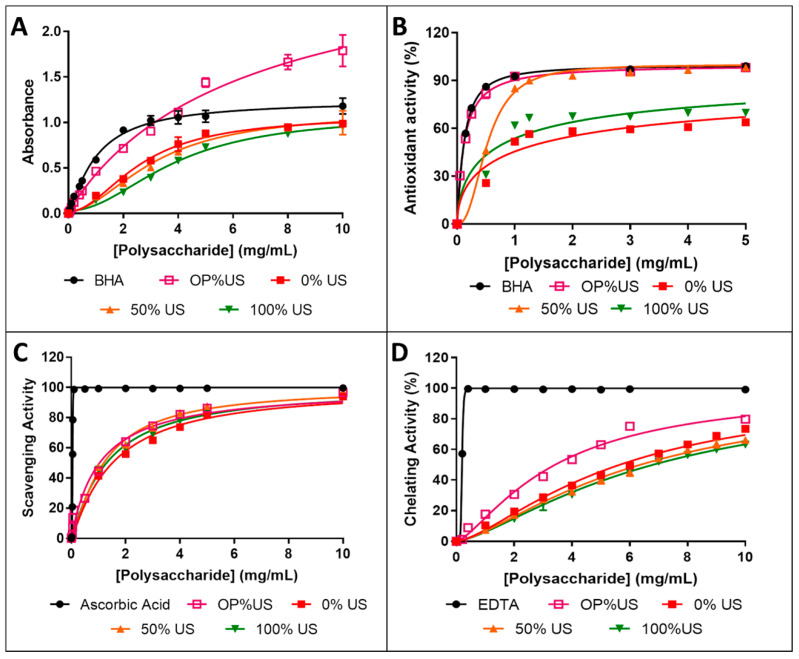
Antioxidant activity of 0, 50, 100% US, and OP%US as a function of concentration in comparison with the control: total antioxidant capacity (**A**), β-carotene bleaching (**B**), ABTS radical scavenging (**C**), metal-chelating power (**D**).

Figure 5A presents the absorbance changes of the four extracts as a function of concentration compared to BHA. The absorbance increases as a function of the concentration from 0.197 to 0.988 for 0% US, from 0.186 to 0.997 for 50% US, and from 0.148 to 0.950 for 100% US, when the concentration increases from 1 to 10 mg/mL. BHA presents higher absorbance with a value of 1.3 at 10 mg/mL. It is of interest that the absorbance of OP%US exceeds that of BHA beyond 4 mg/mL, reaching 1.8 at 10 mg/mL. The comparison of the two combined extraction process at 25 °C for 50% US and 44.2 °C for OP%US indicates that the extraction at the highest temperature increases the reduction capacity of polysaccharides, in agreement with Tran Van Khoa et al. (2019). These authors investigated the effect of different extraction temperatures on the antioxidant activity of polysaccharides extracted from *Ophiocardyceps sobolifera.* The increases of temperature from 60 to 80 °C increases the absorption from 0.13 to 1.9 at 1.5 mg/mL with a very slight increase in polysaccharides yield about 0.49% [38]. Comparison of 0, 50, and 100% US suggests that excessive treatment of ultrasound decreases the absorbance, in agreement with Zhang et al. The authors investigated the effect of different extraction conditions on the antioxidant activity of polysaccharides extracted from *Flammulina velutipes*. No significant difference was detected between the polysaccharides extracted by ultrasound at 30 °C during 40 min with a water to mass ratio of 40 mL/g and those extracted by maceration at 95 °C during 90 min at the same water to mass ratio. The absorbance was approximately 0.2 at 5 mg/mL [39].

Similarly, Hu et al. reported a maximum activity of 0.25 at 350 µg/mL for polysaccharides extracted by ultrasound from *Galla chinensis* at 60 °C, 20 min, and 40 mL/g [40].

#### 3.7.2. β-Carotene Bleaching Inhibition Assay

The capacity of samples to neutralize the linoleic hydroperoxyl radicals is concentration dependent (Figure 5B). The results showed that 50% US presents 98.3% of inhibition at 5 mg/mL, which is very close to those of BHA and OP%US, but much higher than those of 0% US (63.8%) and 100% US (69.8%). It is also of interest to compare the half maximal inhibitory concentration (IC_50_) of the samples. The IC_50_ value is 1.38, 0.52, 0.78, 0.12, and 0.12 mg/mL for 0, 50, 100% US, OP%US, and BHA, respectively. These data imply that all samples have very important inhibitory activity, and that the OP%US presents higher activity than 0, 50, 100% US samples probably because of its higher purity. Nevertheless, no significant difference is noticed between 50% US, OP%US, and BHA in the range from 1.0 to 5.0 mg/mL, which indicates that the use of the combined system at low temperature 25 °C or at optimum extraction temperature 44.2 °C does not affect the capacity of the samples to neutralize the lyophilic radicals. On the other hand, the activity to β-carotene bleaching of 50% US is actually well above that of the 0 and 100% US. The neutralization of linoleic radical R-C=C-(C•H)-C=C-R is based on the capacity of polysaccharide to donate hydrogen. Hernandez-Marin et al. reported that abstraction occurs mostly from C-H rather than from O-H [41]. In our previous work, it was evidenced that the polysaccharides extract is composed of (2→6)-linked β-d-fructofuranosyl (Fruf) backbone with (2→1)-linked β-d-Fruf branched chains [14]. The hydrogen linked to the Carbon 5 of the glucose ring, the hydrogens linked to the Carbon 3 and Carbon 4 of the furanose ring in the backbone, and the hydrogen linked to the Carbon 2 of the terminal group of polysaccharides are accessible sites identified as hydrogen donator and radical neutralization [42,43]. However, the relationships between the antioxidant activity and the structure of polysaccharides is not comprehensively elucidated [44].

Several studies have shown a decrease in the antioxidant activity with increase in molar mass. Xing et al. reported that high molar mass of polysaccharides enhances intramolecular hydrogen bonding, which leads to more compact structure and lower antioxidant and bioactivity [45]. Thus, the high antioxidant activity of the 0, 50, and 100% US samples could be related to their relatively low molar masses (Table 1). Tang et al. reported that the IC_50_ of polysaccharides extracted by ultrasound from *Cyclocarya paliurus* by ultrasound at 25 °C for 20 min with a water to mass ratio of 100 mL/g is 500 μg/mL with a maximum of 59.0%, whereas by maceration the maximum is 55.5% at 1 mg/mL [46].

#### 3.7.3. ABTS Radical Scavenging Activity

The ABTS assay is considered one of the most sensitive techniques to evaluate the antioxidant activity. The inhibition activity increases with increasing concentration to reach a maximum of 94.0, 97.1, 94.0, and 96.4% at 10 mg/mL for 0, 50, 100% US, and OP%US, respectively. In contrast, the inhibition activity of ascorbic acid reaches 100% at 0.1 mg/mL. The IC_50_ value of 50% US is 1.19 ± 0.05 mg/mL, which is slightly lower than those of 0% US (1.51 ± 0.1 mg/mL) and 100% US (1.3 ± 0.1 mg/mL), but is slightly higher than that of OP%US (1.04 ± 0.13 mg/mL) (Figure 5C). The comparison of 50%US and OP%US indicates that the rising of temperature from 25 to 44 °C decreases slightly the IC_50_ about 0.15 mg/mL. Similar results were obtained by Zhang 2018 et al. (2018). They found that the increase in temperature from 30 to 90 °C decreases the IC_50_ about 0.11 mg/mL [47].

The results well agree with literature data. In fact, Hou et al. reported that the IC_50_ values of polysaccharides extracts from Chestnut obtained by hot water and ultrasonic treatments are 3.58 and 1.81 mg/mL, respectively, and suggested that the ultrasound treatment increases the antioxidant activity against ABTS radicals [29]. The scavenging activity of polysaccharides extracted from *Flammulina velutipes* was studied by Hu et al. The maximum activity was 36.7% at a concentration of 3 mg/mL, with an IC_50_ value of 2.8 mg/mL [48]. Chen et al. reported a maximum activity of 34.06% at 1.68 mg/mL in the case of polysaccharides extracted from *Erythronium sibiricum* bulb at 90 °C during 4.28 h with a liquid to mass ratio of 37 mL/g [49]. Comparison of the results with literature indicates that the polysaccharides extracted from *Ornithogalum* have a good scavenging activity against ABTS^+^.

#### 3.7.4. Ferrous Ion-Chelating Activity

Figure 5D presents the ferrous chelating power of the extracts in comparison with EDTA. The results revealed a positive correlation between concentration and chelation activity. A maximum of 73.5 ± 0.1, 66.2 ± 3.5, 63.2 ± 0.1, 79.7, and 99.1% is obtained for 0, 50, 100% US, OP%US, and EDTA at 10 mg/mL, respectively. The five samples present an IC50 value of 5.66 ± 0.08, 6.37± 0.08, 6.8 ± 0.08, 3.4 ± 0.08, and 0.192 ± 0.002 mg/mL. Zhang et al. reported that the polysaccharides extracted using ultrasound exhibit lower metal chelation power than those by maceration. The maximum chelation activity is 38% for ultrasound and 86% for maceration at 5 mg/mL [40]. Similar findings were reported by Surin et al. The authors obtained an IC_50_ of 1.25 and 3.7 mg/mL for polysaccharides extracted by maceration and by ultrasound, respectively [50].

### 3.8. Biocompatibility Evaluation

**Figure 6:**
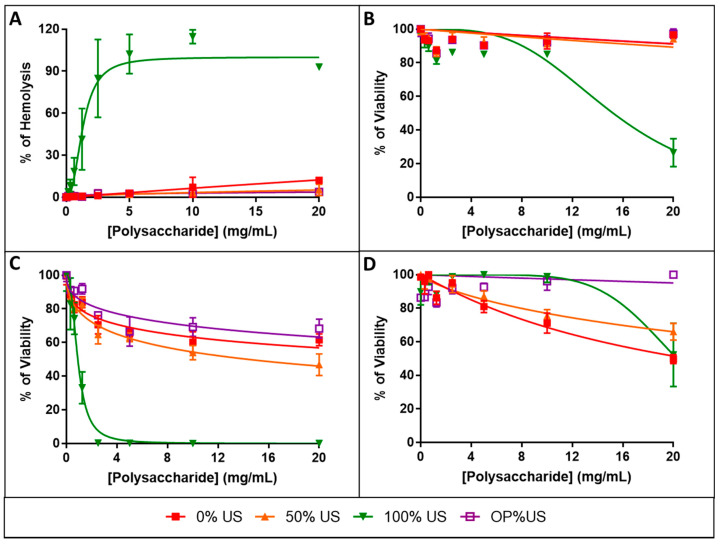
Hemolytic activity of the extracts on human erythrocytes (**A**), toxicity induced by 48 h exposure of human cells HaCaT (**B**), HUVEC (**C**), and IMR90 (**D**) to extracts at increasing concentration. In all cases, results correspond to means ± SD (*n* = 3) and were fitted using the Graph Pad Prism software.

The innocuity of the extracts was evaluated using hemolysis and cytotoxicity assays performed on human cells (Figure 6 and Table 2). Hemolysis of human red blood cells (Figure 6A) was measured after exposure to extracts with increasing concentrations (from 0 to 20 mg/mL, 1:2 serial dilution). Results show that, except 100% US that caused a significant hemolysis with an HC_50_ of 1.32 ± 0.10 mg/mL, 0, 50% US, and OP%US caused none to low membrane leakage of human red blood cells at concentrations up to 20 mg/mL. Cytotoxicity was then measured using normal human cells after 48 h exposure to increasing concentrations of extracts. Tested cells were HaCaT (Figure 6B), HUVEC (Figure 6C), and IMR90 (Figure 6D) corresponding to human normal epithelial skin, vascular endothelial and fibroblastic cells, respectively. Results show that 0, 50% US, and OP%US caused none to low cytotoxicity with IC_50_ values above 20 mg/mL, except for HUVEC cells with an IC_50_ of 14.3 ± 0.2 mg/mL for 50% US. It was found that 100% US was more or less toxic with IC_50_ values of 0.9 ± 0.1, 15.2 ± 1.1 and >20 mg/mL for HUVEC, HaCaT and IMR90 cells, respectively.

### 3.9. Evaluation of Safety Factor

The safety factor (SF) shown in Table 3 is defined as ratio of half maximal hemolytic or cytotoxic concentration to the half maximal inhibitory concentration (HC_50_/IC_50β,_ IC_50_/IC_50β_). The 50% US sample exhibits a SF > 37.04 against RBC, HaCaT, and IMR90 cells, and a lower S.F. of 26.54 for HUVEC cells probably due to the higher sensitivity of the vascular endothelial cells to this extract. The SF values of 50% US sample are higher than those of 0 and 100% US. It is also noticed that the SF of OP%US is >152.7, i.e., much higher than those of 0, 50, and 100% US.

The β-carotene bleaching inhibition assay indicates that the IC_50_ classification (OP%US < 50%US < 100%US < 0%US) is inversely proportional to the polysaccharides percentage (OP%US > 50%US > 100%US > 0%US), which indicates that the antioxidant activity increases with the increases of the polysaccharides%. The results of cytotoxicity for HUVEC cells indicates that the HC_50_ (100%US > 50%US > 0%US ≥ OP%US) follows the same trend of protein percentage (100%US > 50%US > 0%US > OP%US).

These, observations may can be suggested that the SF of OP%US is higher than SF of 50% US owing to the fact that it has the highest purity of polysaccharides, highest antioxidant activity, lowest protein percentage, and lowest cytotoxicity.

## 4. Conclusions

A combined treatment using maceration and sonication allowed getting a vegetal extract from a wild Lebanon plant that has never been studied until now. Three different durations of ultrasound treatment were applied such as the sonication step represents 0, 50, or 100% of the total extraction time of 30 min. The extracts are mainly composed of polysaccharides (from 58.2 to 80.2%) and contain proteins (from 2.4 to 7.7%). The comparison between 50% US extract (30 min, 25 °C, 10 mL/g, 50% US), and OP%US extract (37.1 min, 44.2 °C, 33.8 mL/g, 51.7% US) shows a difference of 3% in the polysaccharide yield. The GC–MS analysis brings new detailed information that indicate that the polysaccharides of the vegetal extracts belong to the family of inulins, neo-inulins, levans, neo-levans, and graminane. TGA, DSC, and SEC analyses did not display significant difference for the three sonication treatments.

The extracts present interesting antioxidant properties. They are able to deactivate lipophilic and hydrophilic radicals. Additionally, the extracts exhibit an inhibition activity for catalyst reaction of radical generation. The best antioxidant was recorded for the beta carotene blanching test for OP%US followed by 50, 100, and 0% US with IC_50_ values 0.131, 0.54, 0.81, and 0.97 mg/mL, respectively. The activity can be therefore enhanced by ultrasonic treatment that improves the purity of the extract when combined to maceration. The complexity of the polysaccharide chains makes it difficult to establish relationships between structure and function, and to further figure out the antioxidant activity of the different extracts. However, it is interesting to note that these extracts have various antioxidant actions according to several mechanisms as depicted by the 4 tests involved in this study. Besides, the results of hemolysis, cytotoxicity, and safety factor indicate that OP%US, 50, and 0% US can be safely used as an antioxidant in food and medical applications. A very outstanding safety factor was found with the extract obtained under the optimal conditions (OP%US) for beta carotene blanching against RBC, HaCaT, HUVEC, and IMR90 cells with SF values higher than 152.7, which could be related to the high polysaccharide’s purity of OP%US. The optimum extraction conditions were found to be at 44.2 °C. It must be emphasized that in the present study, the extracts were obtained at 25 °C, and so with a low energy consumption. These results confirm that the combined process of maceration with sonication either at 25 °C for 50%US or at 44.2 °C for OP%US yields extracts with better properties in terms of antioxidant activity compared to single maceration or ultrasound extraction. Additionally, this study indicates that there is no significant difference between the antioxidant activity of OP%US and 50%US against ABTS and linoleic hydroperoxyl radicals. While, the total antioxidant capacity and metal-chelating power tests show better activities at the highest temperature, highlighting the influence of this parameter.

To conclude, the study on the antioxidant and biological properties of the vegetal extract obtained from this Ornithogalum species from Lebanon revealed the potential of this plant for valuable applications.

## Figures and Tables

**Table 1:** Composition, Molar mass (Mw), and dispersity (Ð) of the extracts.

Extracts	Extraction Yield (%)	Total Carbohydrates Content (%)	Proteins Content (%)	Water Content (%)	Mw	Ð
**0% US**	25.5 ± 4.3	58.2 ±1.1	2.4 ± 0.1	3.4	3140	1.452
**50% US**	45.5 ± 3.2	80.2 ± 1.0	4.8 ± 0.2	3.0	3235	1.467
**100% US**	37.9 ± 2.7	66.1 ± 0.3	7.7 ± 0.3	5.6	3145	1.482
**OP%US**	85.7 ± 0.2%	83.0 ± 0.1%	2.1 ± 0.1	-	-	-

**Table 2:** Half maximal hemolytic concentration (HC_50_) and half maximal cytotoxic concentration (IC_50_) (mg/mL) of the extracts on human cells. HC_50_ and IC_50_ were calculated from Figure 6 using Graph Pad Prism software. Values are expressed as means ± SD.

Test	0% US	50% US	100% US	OP%US
RBC	>20	>20	1.3 ± 0.1	>20
HaCaT	>20	>20	15.2 ± 1.1	>20
HUVEC	>20	14.3 ± 2.0	0.9 ± 0.1	>20
IMR90	>20	>20	>20	>20

**Table 3:** Safety factor (SF) of 0, 50, 100% US, and OP%US based on β-carotene bleaching inhibition test and toxicity tests on human red blood Hemolysis (RBC), HaCaT, HUVEC, and IMR90 cells.

SF	0% US	50% US	100% US	OP%US
HC_50 RBC_/IC_50β_	>20.62	>37.04	1.63	>152.7
IC_50 HaCaT_/IC_50β_	>20.62	>37.04	18.86	>152.7
IC_50 HUVEC_/IC_50β_	>20.62	26.54	1.11	>152.7
IC_50 IMR90_/IC_50β_	>20.62	>37.04	>24.75	>152.7

## Data Availability

All data is presented within the article.

## Supplementary Materials

The following are available online at https://www.mdpi.com/2076-3921/10/1/68/s1, Figure S1: Chromatogram obtained from GC–FID of sucrose after methanolysis and trimethylsilylation, Figure S2: Chromatogram obtained from GC–FID of OP%US of per-trimethylsilylated methyl-glycoside derivatives obtained after methanolysis, Figure S3: Electron impact spectrum of Peak A detected at the elution time of 20 min obtained from sucrose, Figure S4: Electron impact spectrum of Peak B detected at the elution time of 20.5 min obtained from sucrose, Figure S5: Electron impact spectrum of Peak C detected at the elution time 22.3 min obtained from sucrose, Figure S6: Electron impact spectrum of peak D for OP%US sample detected at the elution time of 25.9 min, Figure S7: Electron impact spectrum of peak E for the OP%US sample detected at the elution time of 26.1 min, Figure S8: Electron impact spectrum of peak F for OP%US sample detected at the elution time of 26.3 min, Figure S9: Electron impact spectrum of peaks G for OP%US sample detected at the elution time of 27.6 min.
