# Supplementary material for: Antioxidant Activity and Biocompatibility of Fructo-Polysaccharides Extracted from a Wild Species of Ornithogalum from Lebanon
Source: Antioxidants (Basel). 2021 Jan 7;10(1):68. doi: 10.3390/antiox10010068 (PMC7827233; doi:10.3390/antiox10010068)
Supplement: Supplementary file 1 [file antioxidants-10-00068-s001.pdf]

antioxidants

Article

Antioxidant Activity and Biocompatibility of Fructo-Polysaccharides Extracted from a Wild Species of Ornithogalum from Lebanon

Mohammad Kazem Medlej [1,2], Batoul Cherri [1,2], Hamza Olleik [3], Suming Li [1], Akram Hijazi [2], Ghassan Nasser [2], Marc Maresca [3] and Céline Pochat-Bohatier [1,*]

1 Institut Européen des Membranes, IEM UMR 5635, Université de Montpellier, CNRS, ENSCM, 34095 Montpellier, France; mohammad.medlej1@gmail.com (M.K.M.); batoula.sherry@hotmail.com (B.C.); Suming.Li@umontpellier.fr (S.L.)

2 Platform for Research and Analysis in Environmental Sciences (PRASE), Lebanese University, Beirut, Lebanon; Akram.Hijazi@ul.edu.lb (A.H.); ghassan.nasser@ul.edu.lb (G.N.)

3 Aix Marseille Université, CNRS, Centrale Marseille, iSm2, 13397 Marseille, France; hamza.olleik@live.com (H.O.); m.maresca@univ-amu.fr (M.M.)

* Correspondence: celine.pochat@umontpellier.fr; Tel.: +33-467-143-327

Supplementary Materials: GC-MS analysis

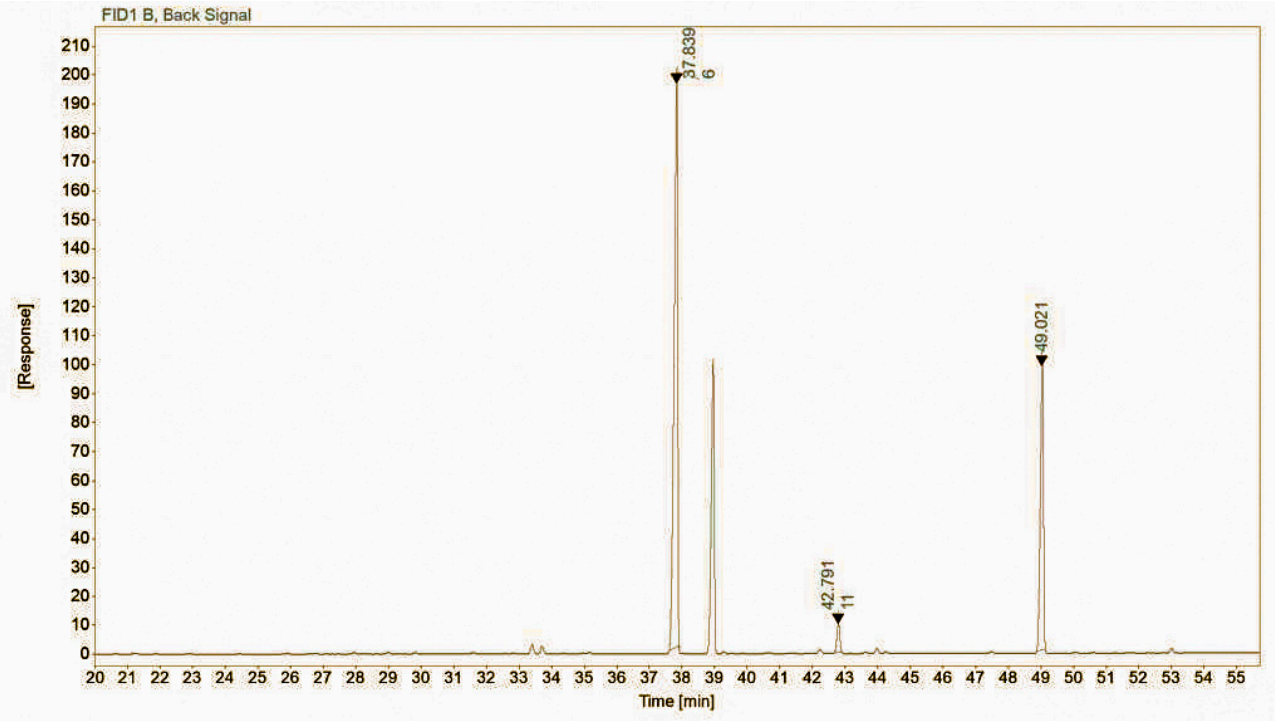

Figure S1. Chromatogram obtained from GC-FID of sucrose after methanolysis and trimethylsilylation (Peak at 49.02 min, internal reference of myoinositol).

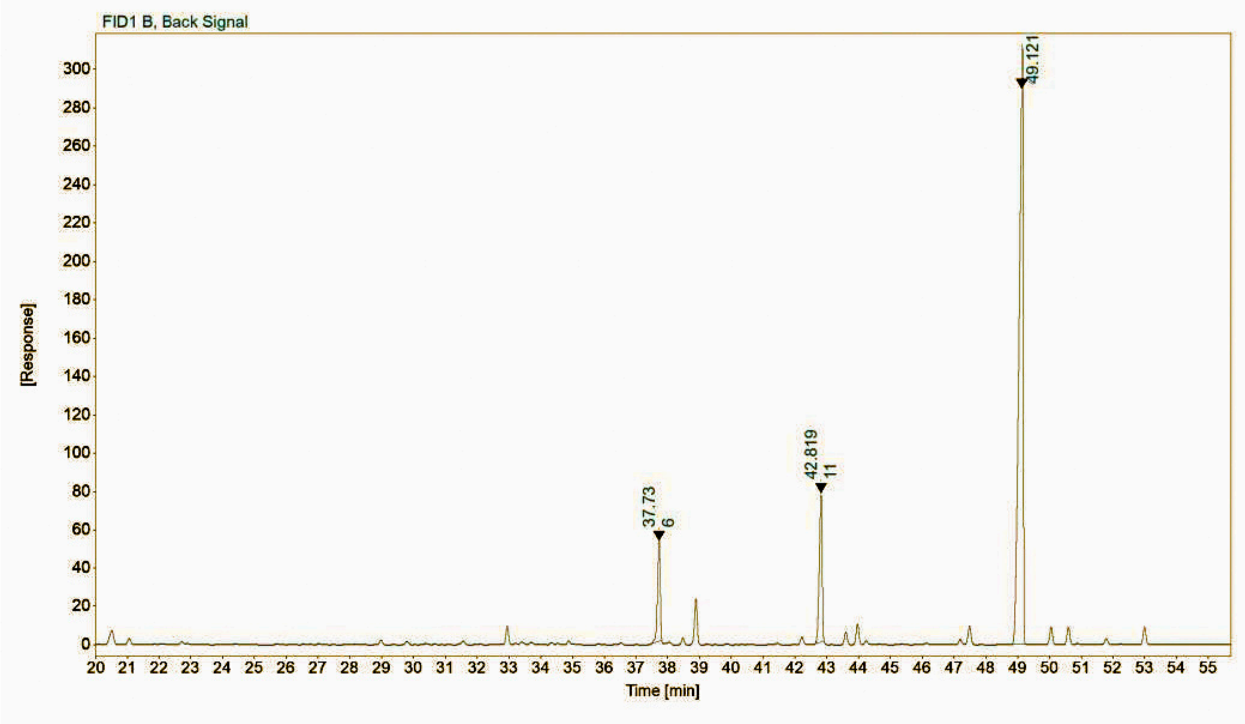

Figure S2. Chromatogram obtained from GC-FID of OP%US of per-trimethylsilylated methyl-glycoside derivatives obtained after methanolysis (peak at 49.12 min, internal reference of myoinositol).

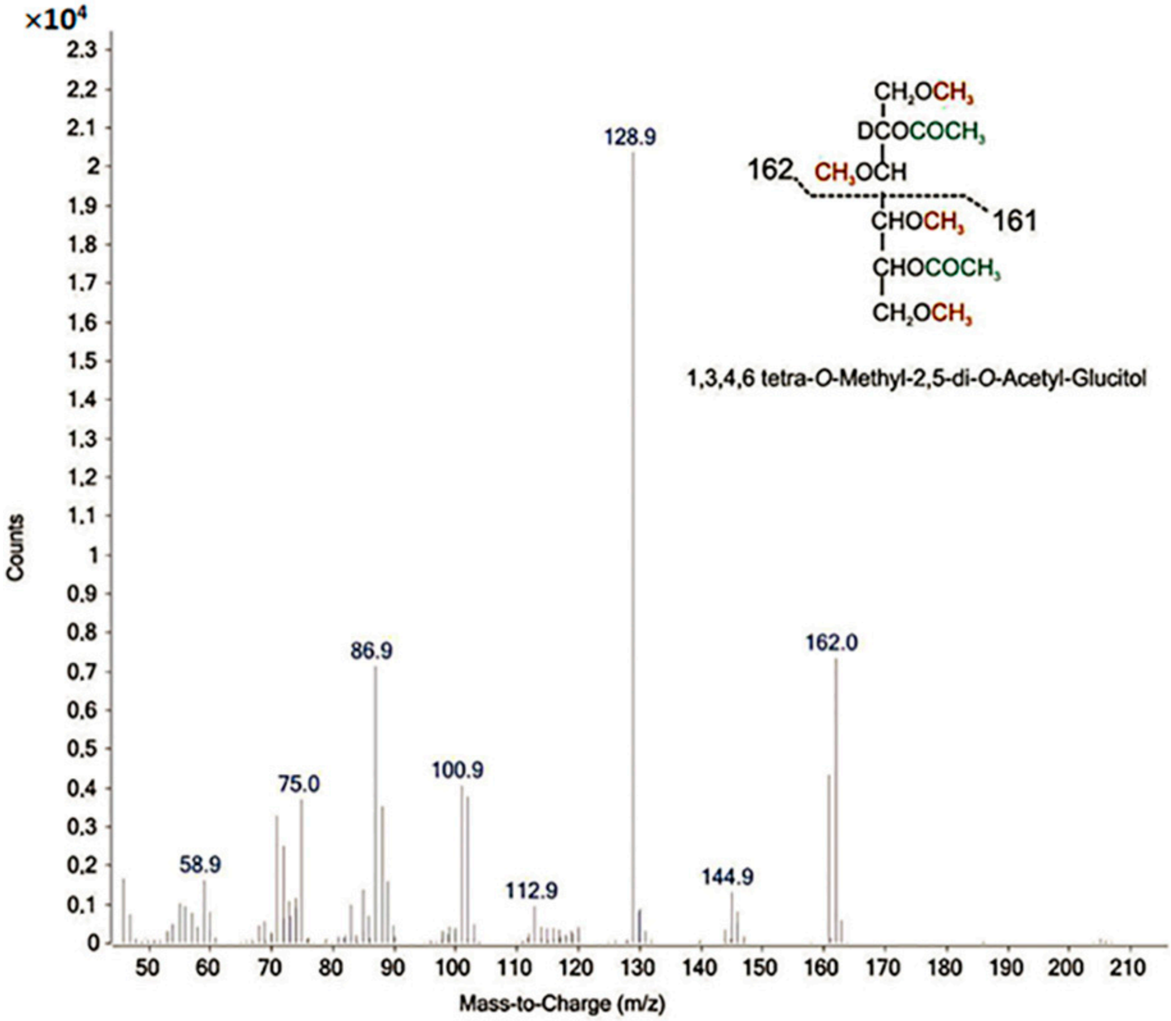

Figure S3. Electron impact spectrum of Peak A detected at the elution time of 20 minutes obtained from sucrose.

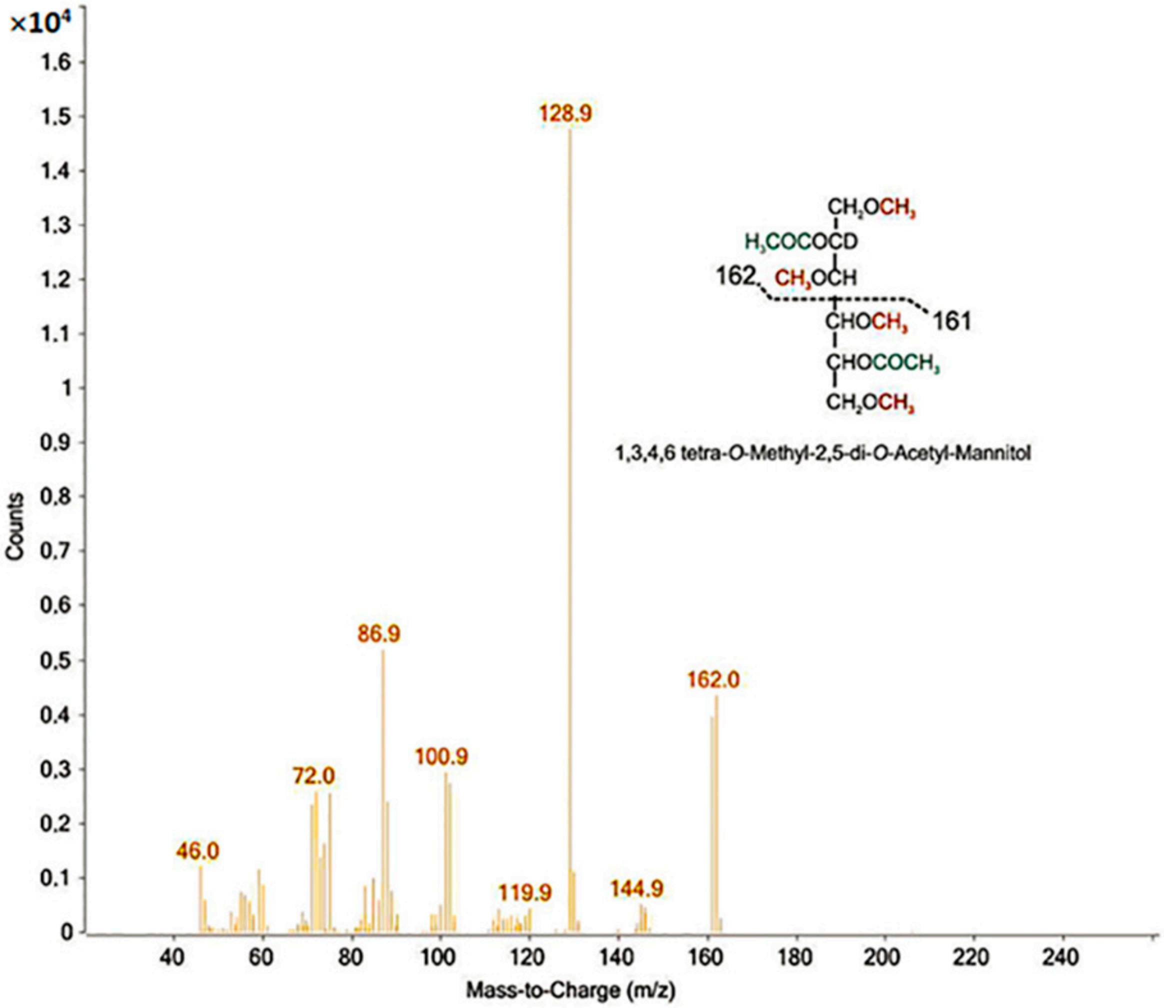

Figure S4. Electron impact spectrum of Peak B detected at the elution time of 20.5 minutes obtained from sucrose.

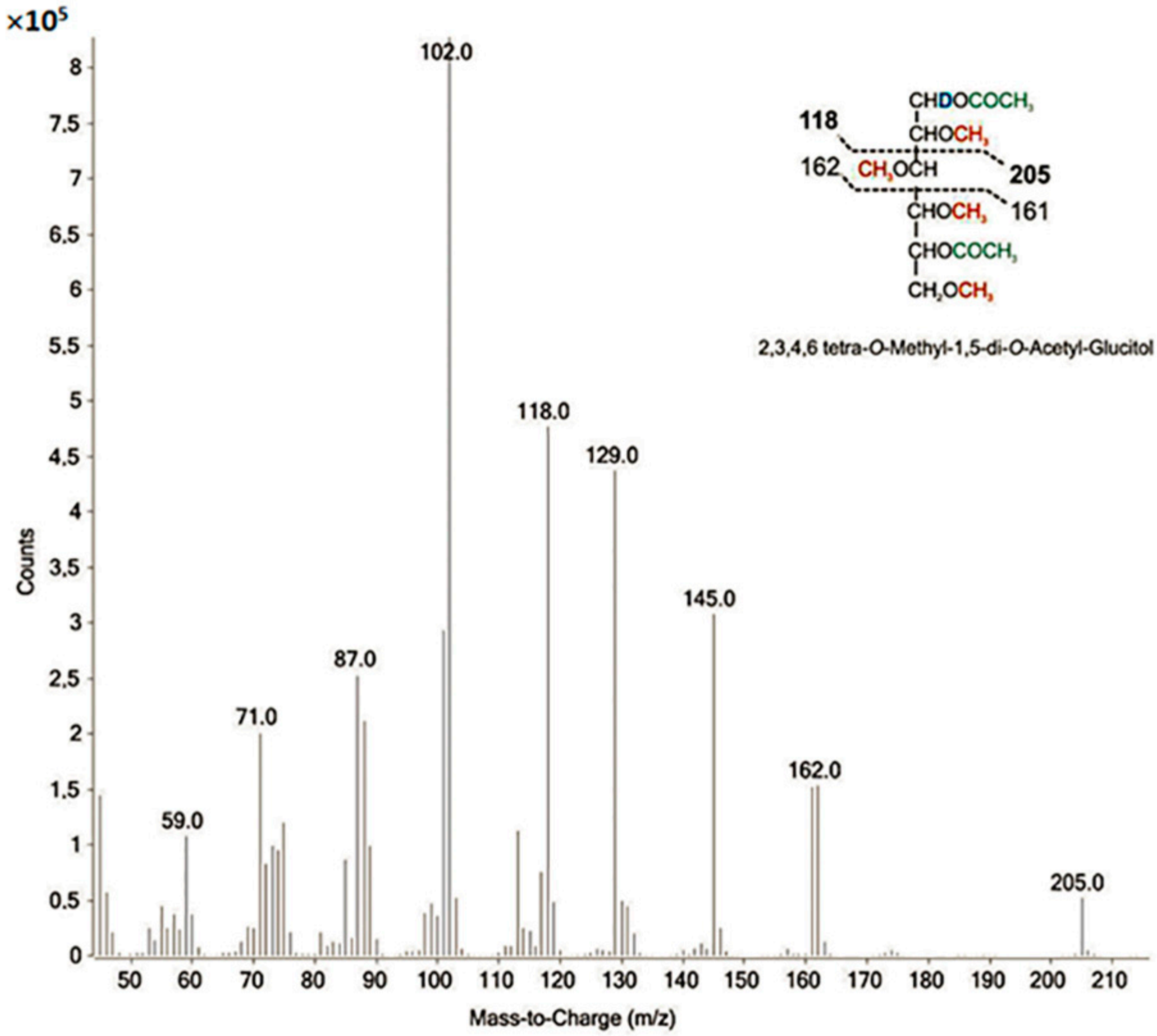

Figure S5. Electron impact spectrum of Peak C detected at the elution time 22.3 minutes obtained from sucrose.

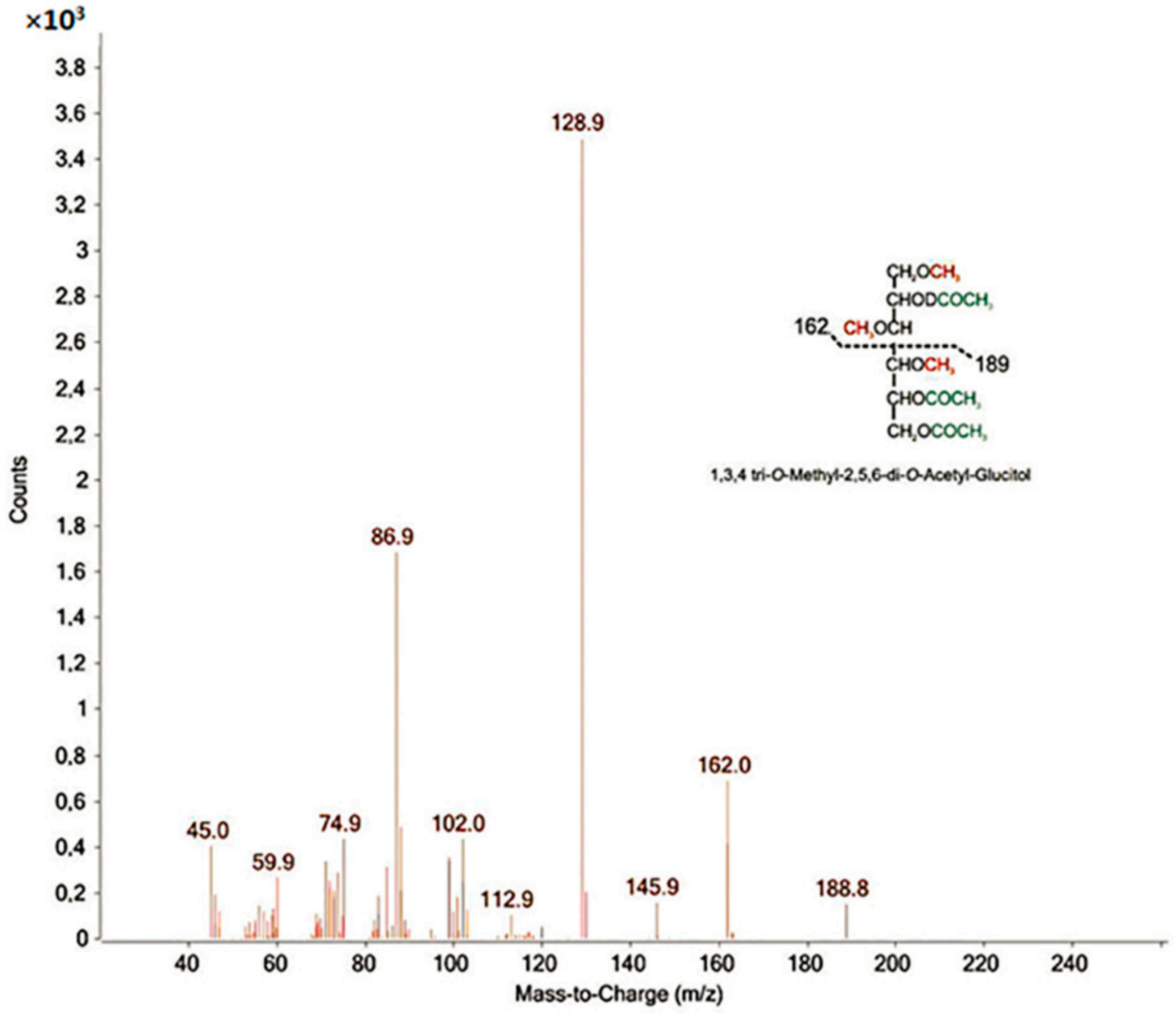

Figure S6. Electron impact spectrum of peak D for OP%US sample detected at the elution time of 25.9 minutes.

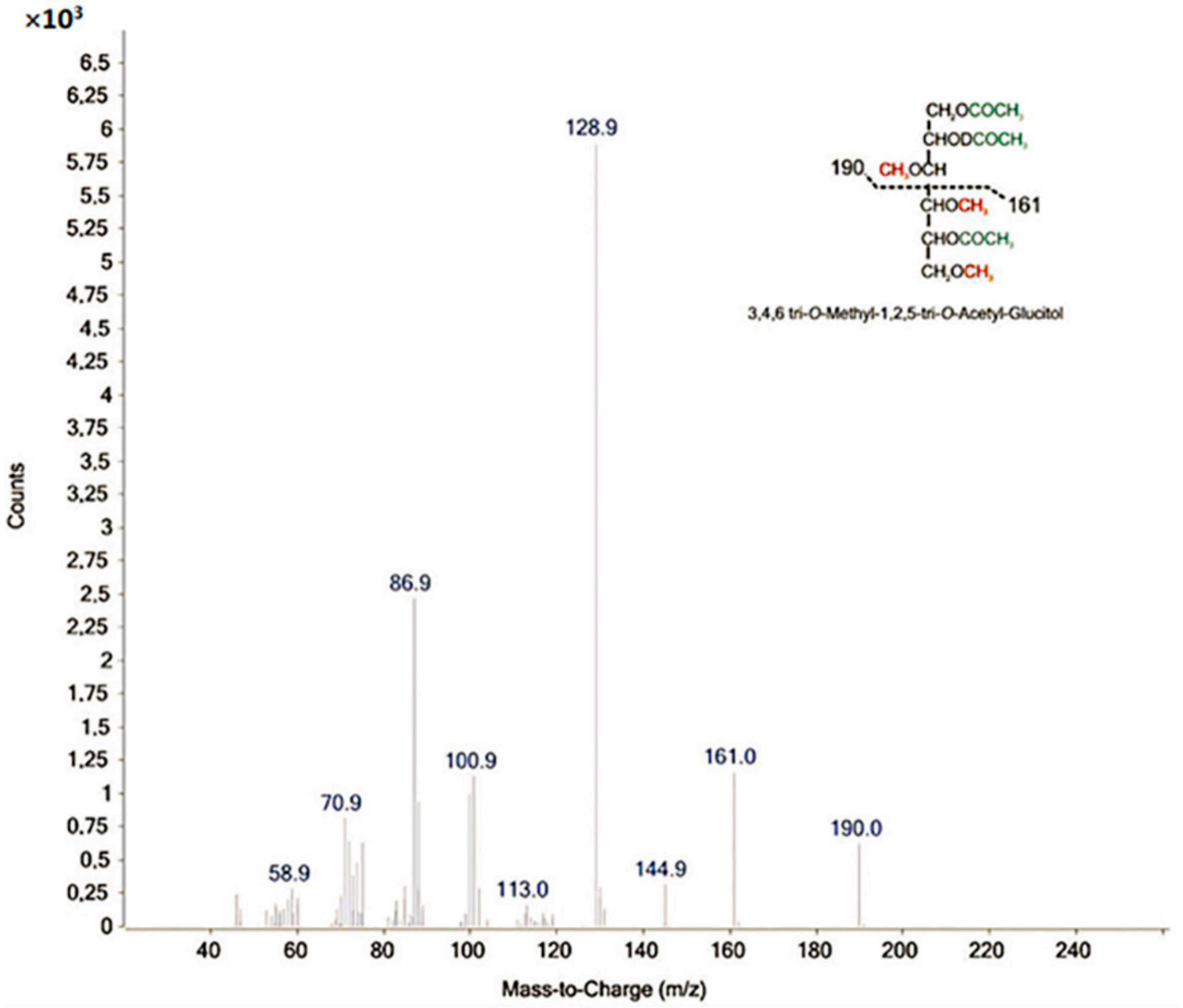

Figure S7. Electron impact spectrum of peak E for the OP%US sample detected at the elution time of 26.1 minutes.

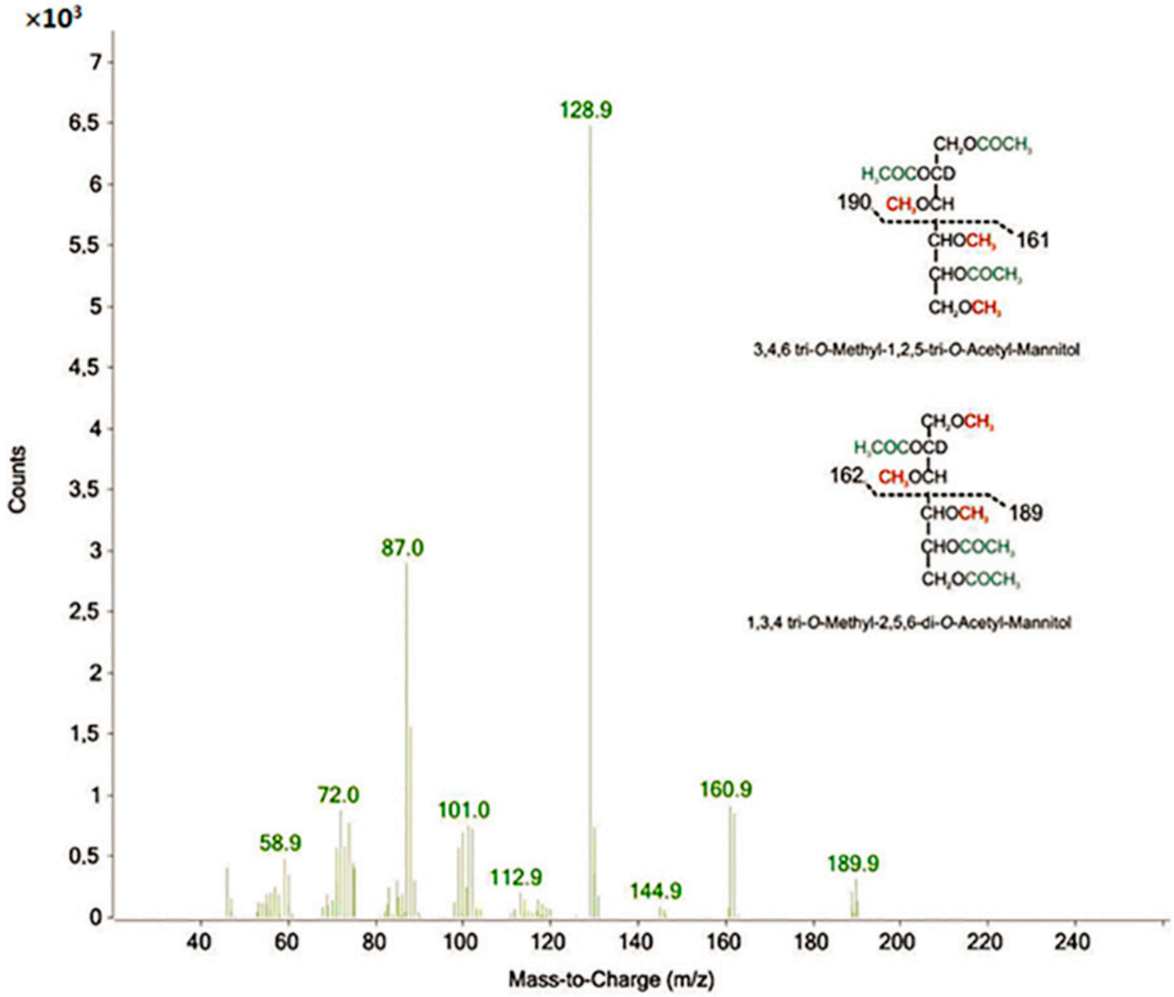

Figure S8. Electron impact spectrum of peak F for OP%US sample detected at the elution time of 26.3 minutes.

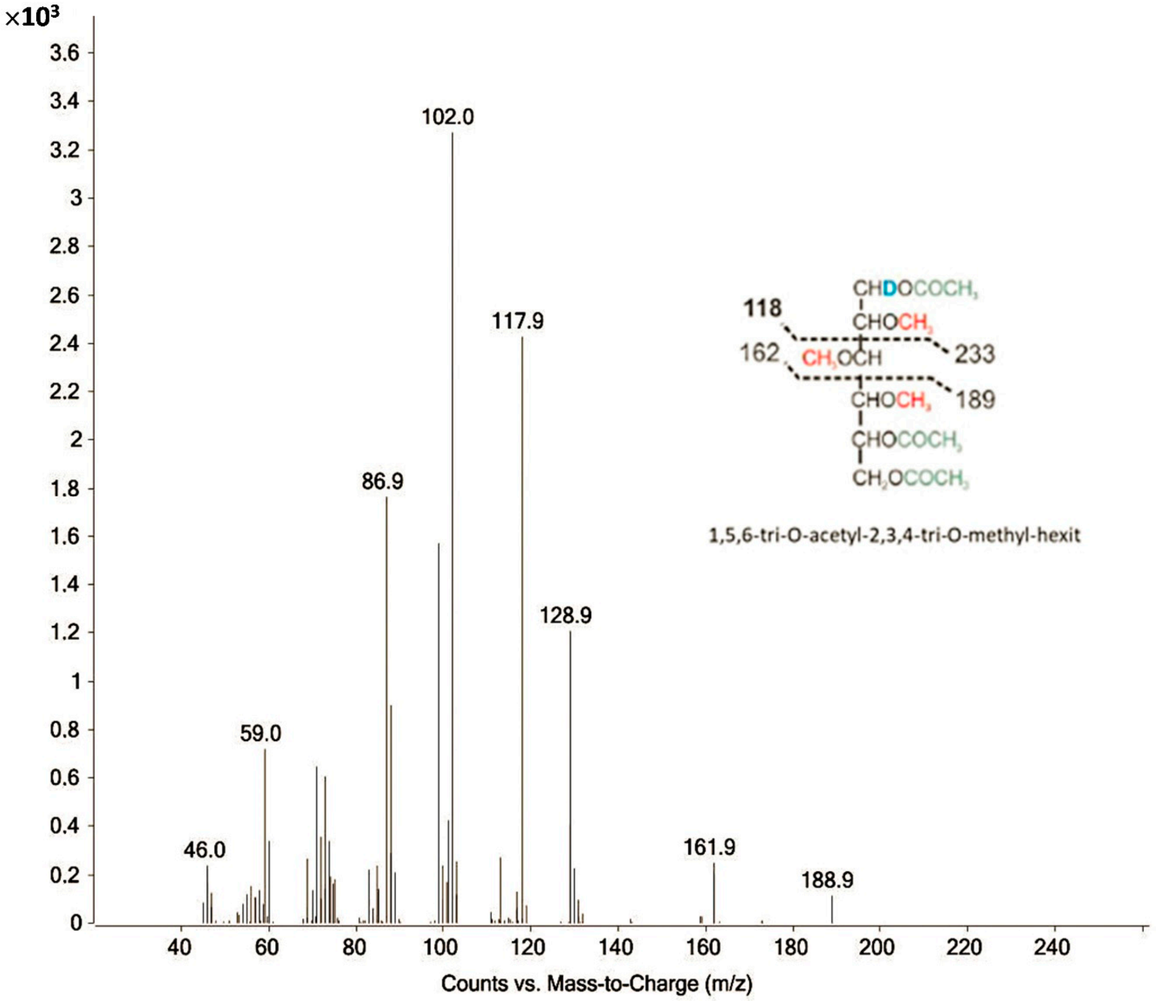

Figure S9. Electron impact spectrum of peaks G for OP%US sample detected at the elution time of 27.6 minutes.

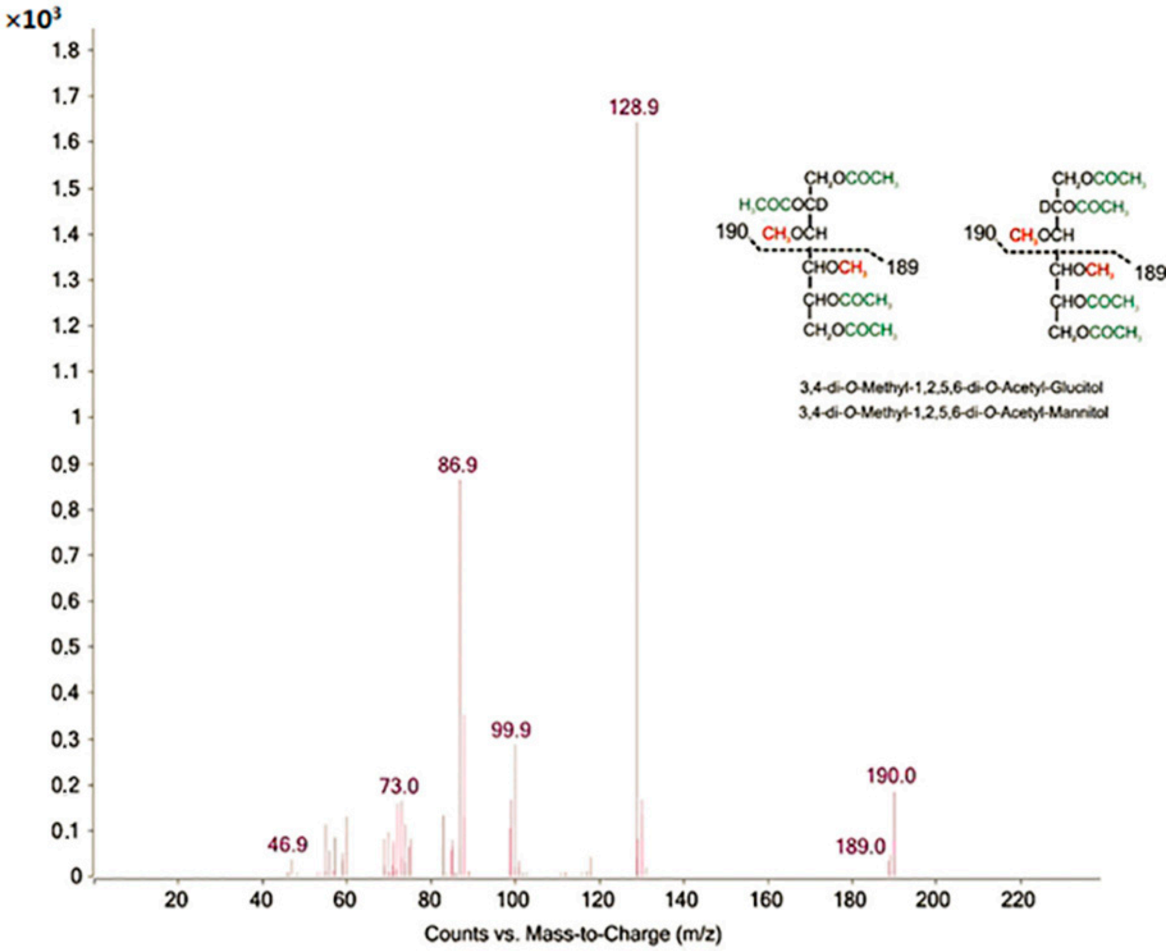

Figure S10. Electron impact spectrum of peaks H and I for OP%US sample detected at the elution time of 31.8 minutes.
